# Comparative efficacy of commercially available deoxynivalenol detoxifying feed additives on growth performance, total tract digestibility of components, and physiological responses in nursery pigs fed diets formulated with naturally contaminated corn^1^

**DOI:** 10.1093/tas/txab050

**Published:** 2021-03-10

**Authors:** Alice W Mwaniki, Quincy R Buis, David Trott, Lee-Anne Huber, Chengbo Yang, Elijah G Kiarie

**Affiliations:** 1 Department of Animal Biosciences, University of Guelph, Guelph, ON N1G 2W1, Canada; 2 Wallenstein Feed & Supply Ltd., Wallenstein, ON, Canada; 3 Department of Animal Science, University of Manitoba, Winnipeg, MB, Canada

**Keywords:** deoxynivalenol, DON detoxifying feed additives, rowth performance, nursery pigs, physiological responses

## Abstract

Comparative efficacy of deoxynivalenol (DON) detoxifying feed additives (FA) was evaluated in growth performance (exp. 1) and apparent total tract digestibility (ATTD; exp. 2) nursery pig studies. Six corn–soybean meal-based diets were used: 1) positive control (PC, formulated with <1.5 ppm DON corn), negative control (NC, formulated with 5.5 ppm DON corn), NC + FA1 (clay plus yeast cell wall extract), NC + FA2 (aluminosilicate), NC + FA3 (aluminosilicate plus fungal extract), and NC + FA4 (sodium metabisulfite, SMB). In exp. 1, 144 pigs (body weight [BW], 10.2 ± 0.1kg) were housed (4 pigs/pen), allocated to diets (*n* = 6) based on BW, and fed for 4-wk. The BW and feed intake were monitored weekly. On d 7, one pig/pen was bled for plasma and euthanized for organ weight and tissue samples. Assayed DON concentration in PC, NC, NC + FA4 was 0.29, 2.86, and 1.21 ppm, respectively. In wk-1, the average daily gain (ADG) of pigs fed NC + FA4 was not different (*P* > 0.05) to that of pigs fed PC diet but greater (*P* = 0.01) than for pigs fed NC without or with other FA. Pigs fed NC and NC + FA2 had lower (*P* = 0.026) average daily feed intake (ADFI) than pigs fed PC and NC + FA3. Pigs fed NC + FA4 had greater (*P* = 0.003) G:F than pigs fed the other diets. Diets had no effect (*P* > 0.05) on ADG, ADFI, and G: F after first week, plasma concentration of urea and creatinine or liver and spleen weight. Pigs fed NC diets had greater (*P* = 0.01) jejunal mRNA expression of superoxide dismutase 1 relative to pigs fed PC or NC plus FA. Jejunal histomorphology and mRNA expression of nutrient transporters, inflammatory cytokines, and tight junction proteins and ceca digesta concentration of short-chain fatty acids were not affected (*P* > 0.05) by the diet. In exp. 2, 24 barrows (BW 10.2 ± 0.3 kg) were individually placed in metabolism crates and allocated to four diets: PC, NC, NC + FA3, and NC + FA4 (*n* = 6) containing TiO_2_ as digestibility marker. Pigs were adjusted to diets for 5 d, followed by a 2-d grab fecal sample collection. Pigs fed PC and NC + FA4 diets had higher ATTD of dry matter, gross energy, and crude protein than NC fed pigs. The FA3 was intermediate in digestibility response. In conclusion, FA containing sequestering component plus fungal extract or SMB in DON-contaminated feed resulted in commensurate nursery pig performance to PC. The tested FA mitigated intestinal oxidative stress through decreased expression of genes for superoxide dismutase.

## INTRODUCTION

Deoxynivalenol (DON) is one of the most common mycotoxins found in cereal grains such as wheat, corn, oat, and barley (Yazar and Omurtag, 2008). The occurrence of DON is associated primarily with *Fusarium graminearum* (*Gibberella zeae*) and *Fusarium culmorum* (Alassane-Kpembi et al., 2015; Mwaniki, 2017). DON is highly water soluble and is effectively absorbed in the upper gastrointestinal tract, that is, stomach, duodenum, proximal jejunum (Dänicke et al., 2004; Goyarts et al., 2006; Broekaert et al., 2017). This mycotoxin has been documented to cause deleterious effects in farm and laboratory animal models (Dillenburger et al., 2001; Broekaert et al., 2017). With the sensitivity order being pigs > mice > rats > poultry = ruminants (Pestka, 2007). It has been suggested that pigs are more sensitive to DON than other farm animals due to differences in the mode of uptake (Bracarense et al., 2012). The major adverse response of ingestion of DON in pigs is reduced feed intake and body weight gain (EFSA Panel on Contaminants in the Food Chain et al., 2017), while acute exposure of high levels may cause emesis (Pestka, 2007). Andretta et al. (2012) carried out a meta-analysis that revealed a reduction of 0.28% in weight gain for each mg per kg of DON in the diet with the effect being greater in younger pigs. Similarly, there was a reduction of 3.9% in the weight gain for each mg per kg of aflatoxin and 0.17% for each mg per kg of fumonisins in the diets (Andretta et al., 2012). Even at low DON doses, co-occurrence with other mycotoxins in naturally contaminated grains might still exert adverse effects on animals due to additive/synergistic interactions among mycotoxins (Goyarts and Dänicke, 2005; Goyarts et al., 2006). Moreover, factors such as dose, duration of exposure, age, sex, and nutrition often determine susceptibility and toxicity to DON (Bryden, 2007; Andretta et al., 2012).

Despite concerted efforts by plant breeders to develop *Fusarium* resistance crops, high prevalence of *Fusarium* damage and DON contamination in cereals is still a major problem in Canada and in other temperate parts of the world (Sabater-Vilar et al., 2007; Mwaniki, 2017; Crippin et al., 2020). Indeed, analyses of feedstuffs and complete feed samples collected from 100 countries from 2008 to 2017 indicated prevalence of DON was 64% in samples from North America (Gruber-Dorninger et al., 2019). Mycotoxins such as DON are very difficult to eliminate once the grains are contaminated because they are heat stable and can withstand feed processing (Hazel and Patel, 2004). To mitigate risks of exposure, the regulatory authorities such as Canadian Food Inspection Agency (CFIA) and Food and Drug Administration (FDA) have mandated maximum level of 1 ppm DON in complete pig feed. Many feed mills have implemented protocols for monitoring and screening feed ingredients to control the amounts of contaminated ingredients used in the feed manufacturing process. However, the presence of multiple mycotoxins at low but varying levels often present risks of chronic ingestion of mycotoxins. Moreover, from feed manufacturing standpoint, monitoring mycotoxins is dependent on the accuracy of methods for sampling and analyses in feedstuffs and complete feed samples. Indeed, several studies have indicated inconsistences in recovery of DON in samples (Patience et al., 2014; Wellington et al., 2020). This has been linked to uneven distribution in the grain due spotted or localized *Fusarium* infestation and the limitations of mixing (Davis et al., 1980).

There are many feed additives (FA) claimed to mitigate the risk of mycotoxicoses in farm animals (Jouany, 2007). However, the majority of these additives are effective in controlling aflatoxin through their adsorption properties (e.g., phyllosilicate minerals, zeolites, activated charcoal, synthetic resins, or yeast cell wall products; Jouany, 2007; Sabater-Vilar et al., 2007). As opposed to aflatoxin, DON is difficult to bind due to its non-polar structure, thus challenging the effectiveness of FA that rely solely on binding capacity (Jouany, 2007; Sabater-Vilar et al., 2007). Various FA have been promoted for enzymatic deactivation, detoxification, and immune support as primary modes of action for mitigating deleterious effects of DON in swine production (Karlovsky, 2011; Patience et al., 2014; Bich et al., 2015a; Frobose et al., 2015; 2017; Tso et al., 2019). However, the empirical data is less convincing. Moreover, there is little information on comparative efficacy of these DON detoxifying additives to record the magnitude of response. Therefore, the overall objective of this study was to evaluate effectiveness of four commercially available FA in mitigating negative effects of feeding DON contaminated diet fed to nursery pigs. To accomplish this objective, two sets of diets were formulated with low (PC) or high (NC) DON corn. Pigs were feed PC or NC without or with select FA to evaluate growth performance and select physiological responses (exp. 1) and apparent total digestibility (ATTD) of components (exp. 2).

## MATERIALS AND METHODS

The experimental protocol (#3786) was reviewed and approved by the University of Guelph Animal Care Committee and pigs were cared for in accordance with the Canadian Council on Animal Care guidelines (CCAC, 2009).

### Experimental Diets and FA

Two batches of corn samples from 2018 growing season were sourced from Wallenstein Feeds & Supply Ltd (Wallenstein, ON, Canada). The two corn batches were categorized as low or high DON based on triplicate mycotoxin panel analyses using LC-MS/MS method in a commercial laboratory (Activation Laboratories, Ancaster, ON, Canada). The low DON corn had 1.45 ± 1.3, 0.11 ± 0.1, 0.23 ± 0.2, 0.10 ± 0.0, 0.06 ± 0.0, 0.06 ± 0.0, and 0.13 + 0.1 ppm of DON, 3-Acetyl-Deoxynivalenol, 15-Acetyl-Deoxynivalenol, Fumonisin B1, T-2, HT-2, and Zearalenone, respectively. The corresponding concentrations in high DON corn were 5.15 ± 0.9, 0.23 ± 0.1, 0.89 ± 0.3, 0.30 ± 0.3, 0.08 ± 0.0, 0.20 ± 0.2, and 0.10 ± 0.0 ppm, respectively. A corn–soybean diet was formulated to meet or exceeded nutrients specifications for nursery pigs (Table 1; NRC, 2012). The corn samples were used to manufacture two sets of diets: a positive control (PC) with low DON corn or negative control (NC) with high DON corn. The NC diet was further split into five portions; four of these portions were top-dressed with four additives based on supplier recommendations. The treated diets were: NC + FA1; Biomin II (Diatomaceous earth, Kaolin, and dehydrated yeast, BIOMIN Canada Inc., Montreal, QC, Canada); NC + FA2; Polaris (Purified hydrated sodium calcium aluminosilicate, Probiotech International, Saint-Hyacinthe, Quebec, Canada); NC + FA3; Epsilon-5 (Hydrated sodium calcium aluminosilicate, shrimp meal, dehydrated *Trichoderma reesei* fermentation extract, Agri-Nutrient Solutions, Innerkip, Ontario, Canada); and NC + FA4; sodium metabisulfite (SMB; Samirian Chemical Inc., Campbell, CA). FA 1–3 were added at a rate of 1 kg/ton and FA4 was added at 3 kg/ton. All diets were prepared in pellet form at a commercial feed mill (Wallenstein Feeds & Supply Ltd., Wallenstein, ON, Canada). To minimize carryover effects, PC diet was manufactured first. The NC diet was mixed in one batch, then split into portions to add FA and subsequently pelleted. The NC diets plus FA were pelleted in numeric order with flushing implemented between each diet. Feed mill worker safety protocols were implemented since SMB liberates sulfur dioxide under hydrothermal conditions such as in the pelleting process. As such personnel were required to wear respirators and safety goggles during manufacturing of NC + FA4.

**Table 1.** Ingredients composition of the basal diet formulation (%, as fed)

Ingredient, %	Amount
Corn	50.0
Soybean meal	29.0
Wheat	14.8
Tallow and poultry fat blend	2.28
L-Lysine	0.43
DL-Methionine	0.22
L-Threonine	0.19
L-Tryptophan	0.01
Lignin sulfonate pellet binder	0.63
Vitamins and trace minerals premix*	2.46
Calculated nutrient content	
Net energy, kcal/kg	2,522
Crude protein, %	19.76
SID Lys, %	1.23
SID Met and Cys, %	0.78
SID Thr, %	0.80
SID Trp, %	0.23
SID Ile, %	0.71
SID Leu, %	1.43
SID Val, %	0.78
Ca, %	0.53
Total P, %	0.61
Digestible P, %	0.29

*Provided per kg of complete feed: vitamin A, 10,000 IU as retinyl acetate; vitamin D3, 1,450 IU as cholecalciferol; vitamin E, 75 IU as dl-α-tocopherol acetate; choline, 250 mg; niacin, 40 mg; pantothenic acid, 19.5 mg; phytase, 500 FTU; Ca, 2.8 g; Cu, 90 mg; Fe, 100 mg; Mn, 52.5 mg; Digestible P,1.3 g; Zn, 150 mg; Na, 2 g; and Se, 0.3 mg (Wallenstein Feeds & Supply Ltd., Wallenstein, ON, Canada).

### Experiment 1

A total of 144 (Yorkshire × Landrace ♀ × Duroc ♂) nursery pigs (72 barrows and 72 gilts), initial body weight (10.2 ± 0.1 kg) were procured from the University of Guelph’s Arkell Swine Research Station (Guelph, ON, Canada). Based on weaning BW, pigs were randomly assigned to pens (4 pigs/pen, 2 barrows, and 2 gilts) in two environmentally controlled rooms. Each room had 12 pens measuring (0.19 m × 4.27 m) equipped with a feeder, a nipple type drinker, plastic-covered expanded metal floors, and partitioning between pens that allowed visual contact with pigs in adjacent pens. The room temperature was maintained at 22°C throughout the experiment. The diets were allocated based randomized complete block design to give six replicates per diet. The number of replicates were deemed adequate based on power analyses using observed variation on average daily gain (ADG) in nursery pigs reared in the test facility (Kiarie et al., 2018). Based on this information, we determined that the six replicates pens will allow detection of 20–25 g/day difference in ADG between pigs fed PC and NC diets at *P* < 0.05 and power of 80%. The pigs were supplied with fresh feed daily and had free access to water for the 4-wk experiment. Body weight and feed intake measurements were determined weekly for calculation of ADG, feed intake (ADFI), and gain-to-feed (G:F). At the end of wk 1, one pig per pen was randomly selected, bled, and sacrificed for organ weight and samples. Blood samples (10 mL) were collected from orbital sinus bleeding technique (Dove and Alworth, 2015) using a Monoject Standard Hypodermic needle 16 G × 1″ (Covidien; Mansfield, MA) into vacutainer tubes coated with lithium heparin (Becton Dickinson & Co, Franklin Lakes, NJ). The samples were immediately centrifuged at 2,000 × *g* for 10 min at 4°C to recover plasma, which was immediately stored at −20°C until used for analyses. Pig was sedated with a premix of 0.2 mL/kg BW (1 mL contained: Ketamine [50 mg], butorphanol [1 mg], and xylazine [10 mg]) via intramuscular injection followed by intravenous injection of Pentobarbital (Euthansol) at 68 mg/kg BW (Kiarie et al., 2020). The spleen and liver were removed, blotted dry with paper towels, weighed, and discarded. Approximately 3 cm of mid jejunum segments were cut and put in buffered formalin for histomorphology analysis. Other segments of mid jejunum (1 cm) were placed in a 2 mL tube filled with 1.2 mL Ambion RNAlater (Life Technologies Inc., Burlington, ON, Canada). The samples were then placed on ice and immediately transported to the lab and stored at −20°C until required for mRNA analyses of digestive enzymes, nutrients transporters, tight junction proteins, and cytokines. Cecum digesta samples were placed in plastic bags and immediately frozen at −20°C for subsequent short-chain fatty acids (SCFA) analyses.

### Experiment 2

A total of 24 barrows with average BW 10.1 ± 0.3 kg were procured from University of Guelph’s Arkell Swine Research Station and housed in individual plexiglass lined pens with tenderfoot floors in a temperature-controlled room (20–22°C). Four of the experimental diets from exp. 1 (PC, NC, NC + A3, and NC + A4) were used for exp. 2. The pelleted diets were ground to mash through a hammermill to allow incorporation 0.20% titanium dioxide as digestible marker. Feeding levels were based on the body weight (2.8 × estimated maintenance energy requirements, NRC, 2012) adjusted for individual pigs at the beginning of each experimental period. Diets were fed as wet mash with a water-to-feed ratio of 2:1 twice daily (0830 and 1630 hours). Water was provided ad lib from a low-pressure drinking nipple. Pigs were adjusted to diets for 5 d, followed by a 2-d grab fecal collection. Fresh fecal samples were collected six times daily, pooled per pig, and stored at −20°C. All pigs were weighed and sacrificed at the end of the experiment for liver and spleen weight as described for exp. 1.

### Sample Processing and Analyses

Samples of the control diets (NC and PC) and NC + FA4 were finely ground and submitted for mycotoxin panel analyses in a commercial laboratory (Activation Laboratories, Ancaster, ON, Canada) as described for corn samples. Another batch of the three ground diets were submitted to a commercial lab (SGS Canada, Guelph, ON, Canada) for dry matter (method 930.15), crude protein (method 968.06, AOAC 2005), crude fat (Method 920.39), crude fiber (Method 978.10), and minerals (method AOAC 968.08) analyses (AOAC, 2005). The stored plasma was used to assay for urea nitrogen and creatinine by photometrics using a Roche Cobas 6000 c501 biochemistry analyzer (Roche Diagnostics USA, Indianapolis, IN) at the Animal Health Laboratory (University of Guelph, Guelph, ON). Fixed jejunal tissues were embedded in paraffin, sectioned (5 μm), and stained with hematoxylin and eosin at the University of Guelph animal health laboratory. In each cross-sectioned tissue, at least 4–5 complete villous-crypt structures were examined under a Leica DMR microscope (Leica Microsystems, Wetzlay, Germany). Villous height and crypt depth were measured using a calibrated micrometer (Kim et al., 2017). Villi height to crypt depth ratio (VH:CD) was calculated. SCFA (lactic, acetic, propionic, and butyric) concentration in ceca samples were analyzed by HPLC (Agilent 1100 Series, Agilent Technologies, Santa Clara, CA; Leung et al., 2018).

Total RNA from 50 to 100 mg of jejunal tissues was extracted using the Trizol method (Thermo Fisher Scientific, Mississauga, ON, Canada) following the manufacturer’s instruction. The RNA was purified by precipitation with lithium chloride and quantified by Nanodrop ND-2000 spectrophotometer (Thermo Fisher Scientific). The ratio of OD260 and OD280 was between 1.8 and 2.1. The integrity of RNA was verified by visualization in an agarose gel. The RNA samples were stored under −80°C. To create a cDNA library, 2 μg of total RNA was reverse transcribed into cDNA using the Superscript II kit (BioRad) following the manufacturer’s instruction. The primers for real-time PCR analysis were designed with Primer-Blast based on the published cDNA sequence in the DNA bank or synthesized based on the primer sequences from publications (Table 2). All the primers spanned at least two exons. The primers were synthesized by Integrated DNA Technologies, Inc. Real-time PCR (RT-PCR) was performed using SYBR Green Supermix (Bio-Rad) on a CFX Connect Real-Time PCR Detection System (Bio-Rad). About 2 μL of cDNA was added to a total volume of 20 μL containing 10 μL SYBR Green mix, and 1 μL each of forward and reverse primers. Each sample was analyzed in duplicate for each gene. The following thermocycling amplification conditions were used: denaturation 15 s at 95°C, annealing 15 s at 56°C, extension 30 s at 72°C, repeating for 40 cycles. A melting curve program was conducted to confirm the specificity of each product. Real-time PCR data were analyzed using the 2^−ΔΔCT^ method to calculate the relative fold change of target gene with GAPDH as internal control (Livak and Schmittgen, 2001).

**Table 2.** Forward and reverse primers for quantitative PCR

Gene	Base pair	Sequence (5′–3′)	Genbank ID	Reference
Glyceraldehyde 3-phosphate dehydrogenase (GAPDH)	89-F	GTGAACGGATTTGGCCGC	NM_001206359.1	Zhao et al. (2019)
	89-R	AAGGGGTCATTGATGGCGAC		
Interleukin-6 (IL-6)	151F	AAGGTGATGCCACCTCAGAC	M86722	Kim et al. (2010)
	151R	TCTGCCAGTACCTCCTTGCT		
Tumor necrosis factor-α (TNF-α)	151F	ATGGATGGGTGGATGAGAAA	X54001	Kim et al. (2010)
	151R	TGGAAACTGTTGGGGAGAAG		
Interleukin-10 (IL-10)	220F	CATCCACTTCCCAACCAGCC	NM_214041	Lee and Kang (2017)
	220R	CTCCCCATCACTCTCTGCCTTC		
Excitatory amino-acid carrier 1 (EAAC1)	168F	CCTCAGTGGTGCTAGGGATTG	NM_001164649.1	Yang et al. (2011)
	168R	GGGCAGCAACACCTGTAATC		
Sodium-dependent neutral amino acid transporter (B0AT1)	154F	AAGGCCCAGTACATGCTCAC	XM_003359855	Yang et al. (2016)
	154R	CATAAATGCCCCTCCACCGT		
Peptide transporter 1(PEPT1)	143F	CATCGCCATACCCTTCTG	NM_214347	Yang et al. (2016)
	143R	TTCCCATCCATCGTGACATT		
Sodium glucose transporter 1 (SGLT1)	153F	GGCTGGACGAAGTATGGTGT	XM_021072101.1	Yang et al. (2011)
	153R	ACAACCACCCAAATCAGAGC		
Zonula occludens-1 (ZO 1)	200F	GATCCTGACCCGGTGTCTGA	XM_021098856	Lee and Kang (2017)
	200R	TTGGTGGGTTTGGTGGGTTG		
Occuludin (OCLN)	163F	GAGAGAGTGGACAGCCCCAT	NM_001163647	Lee and Kang (2017)
	163R	TGCTGCTGTAATGAGGCTGC		
Superoxide dismutase 1 (SOD1)	104F	GTACCAGTGCAGGTCCTCAC	NM_001190422	Lee and Kang (2017)
	104R	TTTGCCAGCAGTCACATTGC		

Fecal samples were thawed overnight in fridge then oven dried at 60°C for 72 h. The samples were subsequently sub sampled, finely ground and along with diets samples analyzed for Ti, DM, nitrogen, and gross energy. Dry matter was determined as described previously and nitrogen analyzed according to Method 990.03 (AOAC, 2005). The CP values were calculated by multiplying analyzed nitrogen values by 6.25. Gross energy was determined using a bomb calorimeter (IKA Calorimeter System C 5000; IKA Works, Wilmington, NC) using benzoic acid as a calibration standard. Titanium concentration was measured using the method of Myers et al. (2004).

### Calculation and Statistical Analyses

Liver and spleen data were expressed relative to BW. The ATTD of components were calculated according to Adeola (2001). The pen was considered the experimental unit in statistical analyses. Experiment 1 data were subjected to GLIMMIX procedures of SAS with block (room) as random effect and diet as fixed effect. Experiment 2 data were subjected to GLIMMIX procedures of SAS with diet as fixed effect. Least square means were separated using Tukey test, and effects were considered significant at *P* ≤ 0.05, and trends (0.051 > *P* ≤ 0.10) were discussed.

## RESULTS

### Experiment 1

The analyzed chemical composition of the control diets and NC + FA4 is shown in Table 3. The NC + FA4 diet was submitted for chemical analyses because it was expected that changes in DON and mineral composition as a result of reaction between SMB and DON could occur under pelleting conditions. Among the assayed mycotoxins, DON predominated with concentration of 0.29, 2.68, and 1.21 ppm in PC, NC, and NC + FA4 samples, respectively. The concentration of sodium in NC + FA4 was 1.3-fold higher than either PC or NC diets and the phosphorous was low in PC by about 0.06% compared with both NC and NC + FA4 diets. The ADG was greater (*P* = 0.01) for pigs fed NC + FA4 compared with pigs fed NC and NC with other FA, but did not differ in pigs fed PC diet (Table 4) in wk 1. As a result, the BW (14.8 kg) of pigs fed NC + FA4 was comparable to that of PC pigs at the end of 1 wk and greater than pigs fed NC and NC with the other FA. Pigs fed NC and NC + FA2 had lower (*P* = 0.026) ADFI than pigs fed PC and NC + FA3, while pigs fed NC + FA2 or FA4 were intermediate. The pigs fed NC + FA4 had greater (*P* = 0.003) G:F than pigs fed PC or NC without or with other FA 1, 2, and 3 in wk 1. With exception of ADFI between d 22 and 28; diets had no effects (*P* > 0.05) on ADG, ADFI, and G:F between d 8 and 28. Pigs fed NC + FA1 had higher (*P* = 0.051) ADFI than pigs fed NC + FA4 and PC diets between d 22 and 28. The final BW was not influenced (*P* > 0.05) by diets. The diets had no effects (*P* > 0.05) on concentration of plasma creatinine and urea, liver and spleen weight, jejunal histomorphology and ceca digesta SCFA after 1 wk of treatment (Table 5). Similarly, diets did not have any effect (*P* > 0.05) on jejunal expression of genes for anti-and pro-inflammatory cytokines, nutrient transporters, and tight junction protein (Table 5). However, pigs fed NC diets showed higher (*P* = 0.01) expression of superoxide dismutase 1 relative to pigs fed PC or NC plus FA. Jejunal expression of superoxide dismutase 1 in pigs fed PC and NC plus FA was not different (*P* > 0.05).

**Table 3.** Analyzed chemical composition of control diets, %, as fed^*^

Item	PC	NC	NC + FA4
Dry matter	86.8	87.2	87.2
Crude protein, %	20.5	20.9	21.0
Crude fat, %	3.28	2.17	2.02
Crude fiber, %	1.99	1.54	1.76
Gross energy, kcal/kg	3,918	3,908	3,901
Ash, %	4.75	4.89	4.79
Calcium, %	0.54	0.60	0.59
Phosphorous, %	0.49	0.55	0.57
Sodium, %	0.27	0.27	0.35
Potassium, %	0.76	0.82	0.86
Magnesium, %	0.13	0.15	0.16
Zinc, ppm	191.3	177.7	168.7
Manganese, ppm	69.2	67.7	65.1
Copper, ppm	104.2	92.0	73.9
Iron, ppm	244.2	270.9	242.5
Mycotoxins			
Aflatoxin B1, ppb	<1.00	<1.00	<1.00
Aflatoxin B2, ppb	<1.00	<1.00	<1.00
Aflatoxin G1, ppb	<1.00	<1.00	<1.00
Aflatoxin G2, ppb	<1.00	<1.00	<1.00
DON, ppm	0.29	2.86	1.21
3-Acetyl-Deoxynivalenol, ppm	<0.06	0.16	0.10
15-Acetyl-Deoxynivalenol, ppm	<0.06	0.38	0.26
Fumonisin B1, ppm	<0.10	<0.10	<0.10
Fumonisin B2, ppm	<0.10	<0.10	<0.10
Ochratoxin A, ppm	<0.003	<0.003	<0.003
T-2, ppm	<0.06	<0.06	<0.06
HT-2, ppm	<0.06	<0.06	0.06
Zearalenone, ppm	<0.03	0.20	0.17
Diacetoxyscirpenol, ppm	<0.03	<0.06	<0.06
Sterigmatocystin, ppm	<0.03	<0.03	<0.03
Mycophenolic acid, ppm	<0.03	<0.03	<0.03

^*^PC, formulated with low corn DON (1.45 ± 1.3 ppm); NC; formulated with high DON corn (5.15 ± 0.93 ppm).

**Table 4.** Effects of supplementation of FA in corn–soybean meal diets contaminated with DON on growth performance of nursery pigs (exp. 1)

	Controls^*^		NC + FA^†^					
Item	PC	NC	FA1	FA2	FA3	FA4	SEM	*P*-value
Body weight, kg								
Initial	10.2	10.5	10.4	10.4	10.2	10.2	0.105	0.379
Day 7	14.5^ab^	14.0^b^	14.2^b^	14.0^b^	14.2^b^	14.8^a^	0.167	0.016
Day 14	20.0	19.3	19.1	19.2	19.4	20.3	0.287	0.142
Day 21	25.3	25.2	24.4	25.1	25.3	25.9	0.369	0.147
Day 28	31.3	30.8	30.4	30.8	31.1	32.2	0.463	0.221
ADG, g/day								
Day 0–7	608^ab^	511^c^	551^bc^	517^c^	583^b^	655^a^	20.12	0.010
Day 8–14	800	753	698	750	743	782	31.17	0.523
Day 15–21	761	847	750	846	839	804	38.91	0.355
Day 22–28	853	792	863	804	831	893	38.19	0.530
Day 0–28	752	725	717	728	748	784	17.56	0.119
ADFI, g/day								
Day 0–7	829^a^	728^b^	772^ab^	726^b^	809^a^	776^ab^	23.65	0.026
Day 8–14	1,175	1,144	1,085	1,123	1,137	1,176	32.17	0.369
Day 15–21	1,405	1,331	1,325	1,318	1,331	1,395	66.13	0.881
Day 22–28	1,695^b^	1,725^ab^	1,923^a^	1,594^b^	1,780^ab^	1,591^b^	77.41	0.051
Day 0–28	1,276	1,232	1,276	1,190	1,264	1,235	35.48	0.506
G: F, g/g								
Day 0–7	0.734^b^	0.704^b^	0.719^b^	0.712^b^	0.725^b^	0.845^a^	0.024	0.003
Day 8–14	0.681	0.639	0.644	0.679	0.659	0.638	0.024	0.647
Day 15–21	0.550	0.637	0.574	0.643	0.630	0.602	0.039	0.490
Day 22–28	0.505	0.464	0.456	0.506	0.471	0.577	0.031	0.096
Day 0–28	0.590	0.590	0.566	0.613	0.593	0.643	0.019	0.140

^*^PC, formulated with low corn DON (1.45 ± 1.3 ppm); NC; formulated with high DON corn (5.15 ± 0.93 ppm).

^†^FA1; Biomin II (Diatomaceous earth, Kaolin, and dehydrated yeast, BIOMIN Canada Inc., Montreal, QC, Canada); FA2; Polaris (Purified hydrated sodium calcium aluminosilicate, Probiotech International, Saint-Hyacinthe, QC, Canada); FA3; Epsilon-5 (Hydrated sodium calcium aluminosilicate, shrimp meal, dehydrated *Trichoderma reesei* fermentation extract dehydrated, Agri-Nutrient Solutions, Innerkip, ON, Canada); and FA4; Sodium metabisulphite (Samirian Chemical Inc., Campbell, CA).

Within a row, means with different superscripts, differ, *P* < 0.05 (*n* = 6).

**Table 5.** Effects of supplementation of FA in corn–soybean meal diets contaminated with DON on plasma metabolites, organ weight, and gastrointestinal measurements in nursery pigs fed diets (exp. 1)^*^

	Controls^†^		NC + FA^‡^					
Item	PC	NC	FA1	FA2	FA3	FA4	SEM	*P*-value
Plasma metabolites								
Creatinine, µmol/L	49.3	49.2	50.5	49.9	49.7	49.3	2.13	0.995
Urea, mmol/L	1.27	1.48	1.02	1.27	1.38	1.6	0.25	0.683
Organ weight								
Body weight (BW), kg	14.7	13.7	14.1	14.3	14.7	14.4	0.50	0.676
Liver, g	446	461	470	422	470	436	25.6	0.708
Liver, g/kg BW	30.4	33.7	33.3	29.7	31.9	30.4	1.55	0.336
Spleen, g	27.6	28.3	25.5	27.9	27.3	32.8	1.96	0.197
Spleen, g/kg BW	1.88	2.08	1.81	1.95	1.86	2.29	0.14	0.154
Jejunal histomorphology								
Villi height (VH), µm	949	1,010	941	940	1,063	981	67.9	0.761
Crypt depth (CD), µm	373	400	389	407	388	380	22.1	0.885
VH: CD ratio	2.6	2.6	2.4	2.4	2.8	2.6	0.20	0.722
Jejunal mucosal gene expression								
Tumor necrosis factor-α	1.71	1.44	1.94	2.23	2.84	2.86	0.84	0.778
Interleukin-6	2.97	3.28	2.36	1.93	4.20	3.88	0.91	0.486
Interleukin-10	3.04	2.99	3.45	2.91	4.16	2.27	0.78	0.664
Sodium-dependent neutral amino acid transporter	3.28	4.17	3.18	3.03	2.70	4.77	1.16	0.805
Excitatory amino-acid carrier 1	2.05	2.42	2.51	1.83	2.95	4.26	0.84	0.389
Peptide transporter 1	5.61	6.09	5.11	5.21	5.09	7.07	1.75	0.961
Sodium glucose transporter 1	4.41	8.11	6.13	6.32	5.05	7.98	2.06	0.738
Occuludin	14.6	16.7	14.2	10.3	16.2	17.5	3.66	0.778
Zonula occludens-1	3.89	4.93	3.76	3.85	5.87	5.05	0.86	0.438
Superoxide dismutase 1	5.04^b^	16.7^a^	4.35^b^	4.16^b^	6.48^b^	6.85^b^	1.50	0.001
Ceca digesta SCFA^||^, µmol/g								
Lactic	10.9	3.31	12.7	6.38	25.6	2.68	8.05	0.291
Acetic	42.4	57.4	49.8	45.6	48.0	57.1	5.27	0.283
Propionic	22.5	26.9	26.2	24.5	26.0	24.6	2.25	0.767
Butyric	16.9	18.4	14.2	13.2	17.3	13.2	3.41	0.821

^*^Samples collected after one-week experimentation.

^†^PC, formulated with low corn DON (1.45 ± 1.3 ppm); NC; formulated with high DON corn (5.15 ± 0.93 ppm).

^‡^FA1; Biomin II (Diatomaceous earth, Kaolin, and dehydrated yeast, BIOMIN Canada Inc., Montreal, QC, Canada); FA2; Polaris (purified hydrated sodium calcium aluminosilicate, Probiotech International, Saint-Hyacinthe, QC, Canada); FA3; Epsilon-5 (Hydrated sodium calcium aluminosilicate, shrimp meal, dehydrated *Trichoderma reesei* fermentation extract dehydrated, Agri-Nutrient Solutions, Innerkip, ON, Canada); and FA4; Sodium metabisulphite (Samirian Chemical Inc., Campbell, CA).

^||^Short-chain fatty acids.

Within a row, means with different superscripts, differ, *P* < 0.05 (*n* = 6).

### Experiment 2

There were no (*P* > 0.05) diet effects on BW, ADG, liver, and spleen weights (Table 6). Pigs fed PC and NC + FA4 diets had greater ATTD of dry matter, gross energy, and crude protein than NC fed pigs (*P* < 0.05), whereas pigs fed NC + FA3 had intermediate ATTD values (Table 6).

**Table 6.** Effects of supplementation of FA in corn–soybean meal diets contaminated with DON on body weight, organ weight, and ATTD of components in nursery pigs (exp. 2)

	Controls^*^		NC + feed addtives^†^			
Item	PC	NC	FA3	FA4	SEM	*P*-value
Initial BW, kg	10.1	10.2	10.1	10.3	0.30	0.986
Final BW, kg	12.0	12.1	12.0	12.1	0.23	0.999
ADG, g/day	264	273	273	254	30.9	0.967
Organ weight						
Liver, g	306	296	305	297	12.6	0.912
Liver, g/kg BW	25.5	24.5	25.3	24.7	1.00	0.863
Spleen, g	29.9	28.8	28.5	25.8	2.29	0.665
Spleen, g/kg BW	2.52	2.37	2.36	2.15	0.19	0.620
ATTD, %						
Dry matter	83.7^a^	77.3^b^	81.5^ab^	84.1^a^	2.12	0.043
Gross energy	82.0^a^	74.2^b^	79.0^ab^	82.2^a^	2.52	0.042
Crude protein	75.7^a^	63.8^b^	68.2^ab^	74.7^a^	3.86	0.027

^*^PC, formulated with low corn DON (1.45 ± 1.3 ppm); NC; formulated with high DON corn (5.15 ± 0.93 ppm).

^†^FA3; Epsilon-5 (Hydrated sodium calcium aluminosilicate, shrimp meal, dehydrated *Trichoderma reesei* fermentation extract dehydrated, Agri-Nutrient Solutions, Innerkip, ON, Canada) and FA4; Sodium metabisulphite.

Within a row, means with different superscripts, differ, *P* < 0.05 (*n* = 6).

## DISCUSSION

Ingestion of DON at relatively low concentrations (0.75–3.0 ppm) from naturally contaminated feedstuffs has been shown to reduce BW gain and feed intake in nursery pigs (Rotter et al., 1994; Holanda and Kim, 2020). We used corn samples naturally contaminated with DON to create a low and high DON-contaminated feed (0.29 vs. 2.86 ppm) for nursery rations as a basis for evaluating efficacy of FA. Although we are attributing pig responses to DON and its metabolites (e.g., 15-ADON, 3-Acetyl DON), we cannot preclude impact of other mycotoxins in the corn sample even though they were detected at lower levels. The decrease in assayed DON in NC + FA4 relative to NC was attributed to the formation of DON-sulfonate, a nontoxic product formed by the reaction between SMB and DON during hydrothermal conditions of pelleting (Dänicke et al., 2005; Frobose et al., 2017). Pigs fed NC diet exhibited 16% and 12% reduction in ADG and ADFI relative to PC pigs after 7 days of exposure. However, there was no DON effect on ADG and ADFI after d 8. We also did not observe differences between PC and NC on ADG in exp. 2. Nursery pigs (~10 kg) fed corn-based diets with 0.75, 1.50, and 3.0 ppm DON had 8%, 19%, and 25%, respectively, had reduced ADG relative to control (0.0 ppm) in the first 7 days exposure but not after d 8 in a 28-d trial (Rotter et al., 1994). However, the negative effects on ADFI was observed through to d 28 but with decreasing magnitude (Rotter et al., 1994). Several other studies using >3 ppm DON have reported progressive disappearance or blunting of negative effects of DON on growth in nursery (Pollmann et al., 1985; Bich et al., 2015a; Frobose et al., 2015) and grow-finish pigs (Foster et al., 1986; Patience et al., 2014; Serviento et al., 2018; Wellington et al., 2020). It has been demonstrated through meta-analyses that reduction in ADG in pigs due to mycotoxicosis was highly correlated with depression in feed intake and feed efficiency (Pastorelli et al., 2012). Indeed, feed efficiency response to DON in pigs is inconsistent. For example, some studies reported no (Wellington et al., 2020), negative (Bich et al., 2015a; Frobose et al., 2015), or positive (Rotter et al., 1994) effects.

Several commercially available FA that claim to modulate negative effects of DON in pigs were evaluated in the present study. The additives were selected based on availability in Ontario and had different components, FA1–FA3 had sequestering agents without (FA1 and FA2) or with fungal metabolites (FA3) whereas FA4 was SMB, a DON detoxifier (Jouany, 2007; Sabater-Vilar et al., 2007; Bich et al., 2015a). Sequestering agents such as aluminosilicates, bentonites, yeast well walls are postulated to bind mycotoxins to reduce or prevent their toxic effects upon ingestion (Jouany, 2007). Flow agents and pellet binders may also have mycotoxin sequestering effects (Frobose et al., 2015; Tilley et al., 2017). Given the observed growth performance differences between PC and NC in the first week, it is plausible that the pellet binder used in the present study had no DON sequestration effect. Mycotoxin binding agents such as clay and aluminosilicates have been reported to be less effective in mitigating negative effects of DON on growth performance in pigs (Döll et al., 2005; Bich et al., 2015a). These observations seemed to have been confirmed in the present study with respect to FA1 and FA2. However, pigs fed NC + FA3 that contained aluminosilicates, shrimp meal, and fungal extract exhibited better ADG and ADFI than NC pigs in wk 1, but no other benefit relative to the NC. Although not analyzed, it is plausible the metabolites such as enzymes and other functional metabolites from fungal extract may have influenced pig growth, as has been shown previously (Bich et al., 2015a). FA4 resulted in commensurate ADG and ADFI to PC and superior G:F than all diets in wk 1. Similarly, adding SMB to a diet with 4 ppm DON resulted better ADG and G:F compared with the PC diet (0.05 ppm; Frobose et al., 2017). Several other studies have also shown that FA containing SMB mitigated negative effects of DON growth performance in pigs (Patience et al., 2014; Bich et al., 2015a; Frobose et al., 2015).

Ingested DON has been reported to be rapidly absorbed in the GIT increasing risks of systemic toxicity and organ damage (Williams et al., 1988; Richard, 2007; Chen et al., 2008). Studies in pigs have focused on accumulation of DON in the kidney and liver tissues, organ atrophy, and altered metabolism (Pollmann et al., 1985). Moreover, DON can impair liver and kidney function resulting in increased plasma creatinine and blood urea nitrogen concentrations (Bryden, 2007; Maresca and Fantini, 2010). DON-induced liver, spleen and kidney atrophy, and altered metabolism in pigs has been linked to depressed appetite and systemic toxicity (Pollmann et al., 1985; Rotter et al., 1994; Swamy et al., 2002). Although we observed depressed ADFI in NC pigs, this did not have bearing on liver and spleen weights or concentration of plasma creatinine and urea nitrogen. Similarly, 0.75–3.0 ppm DON had no effect on liver and spleen weight and plasma creatinine and urea nitrogen in nursery pigs on DON contaminated feeds mixed with yeast-based additive (Rotter et al., 1994; Holanda and Kim, 2020). However, studies that fed higher level of DON >3.5 ppm reported reduced liver and spleen weight and increased plasma creatinine and urea in pigs (Pollmann et al., 1985; Swamy et al., 2002; Wu et al., 2015).

After DON contaminated feed is ingested, the intestinal tract is the first organ that is affected, which may lead to intestinal epithelial cells being exposed to high levels of mycotoxin (Grenier and Applegate, 2013; Ghareeb et al., 2015; Pierron et al., 2016). Mycotoxins, including DON and its metabolites, are known to alter GIT ecology and physiology (Suzuki and Iwahashi, 2015; Robert et al., 2017). Previous studies indicated pigs fed 3–12 ppm DON had shorter villi and decreased expression of nutrient transporters than control pigs (<0.5 ppm DON; Bracarense et al., 2012; Bich et al., 2015a; Wu et al., 2015). However, the level of DON employed in the present study did not influence jejunal histomorphology, mRNA expression of nutrient transporters, pro-inflammatory cytokines, and tight junction proteins. Adverse effects of DON on intestinal tissue is linked with increased production of free radicals and reactive oxygen species (ROS), resulting in oxidative damage to enterocytes (Kouadio et al., 2005; Li et al., 2014; Bich et al., 2015b). Exposure of mice colon cells to DON increased superoxide dismutase activity in concomitant with elevation of ROS (Krishnaswamy et al., 2010). Increased concentration of intestinal malondialdehydes and decrease in glutathione peroxidase in pigs fed DON contaminated diets has been reported as an indicator for jejunal mucosa oxidative stress (Bich et al., 2015b; Holanda and Kim, 2020). In the present study, pigs fed NC diet exhibited higher mRNA expression of superoxide dismutase relative to pigs fed PC or NC with FA. Lower mRNA expression of superoxide dismutase in pigs fed FA might suggest mitigation of DON-induced oxidative stress at the intestinal level. It has also been argued that some microbial components in these FA, such as glucomannans, may directly ameliorate deleterious effects of DON at the cellular level (Bich et al., 2015b; Holanda and Kim, 2020). Investigations on the impact of DON on intestinal microflora activity is rarely reported, yet the toxin impact of digestive capacity will alter the flow undigested material in the hindgut. We did not observe changes in concentration of cecal digesta SCFA in the present study. However, consumption of DON (2.8 mg/kg) contaminated feed reduced pig growth performance and had a moderate effect on cultivable intestinal bacteria (Waché et al., 2009).

Pigs fed PC and NC + FA4 had higher ATTD of energy and protein than NC pigs fed pigs. Pigs fed 3 ppm DON showed lower dry matter, energy, and fat digestibility than control (<0.05 ppm) fed pigs (Bich et al., 2015a). Other studies reported increased nutrient digestibility in pigs fed DON-contaminated diets (Dänicke et al., 2004; Goyarts and Dänicke, 2005). This has been linked to physiologic adaptation to decreased feed intake or *Fusarium* fungi facilitated the digestion of cereal components by monogastrics via partial cell wall degradation (Dänicke et al., 2004). However, the inclusion of wheat and fat source for energy lowered corn inclusion rate and may have limited the ability to find a bigger response.

DON from naturally contaminated corn caused reduction in growth performance and nutrient digestibility and induced intestinal oxidative stress within the first 7 days of exposure. The disappearance of deleterious effects of DON on growth performance after a short feeding extended previous observations suggesting tolerance. Arguably, this may not be necessarily the case in commercial pork production system largely because pigs will often receive feed made from different batches of feedstuffs presenting a scenario of exposure of multiple mycotoxins at varying levels. The use of FA containing sequestering components in DON-contaminated diets were ineffective on ameliorating growth performance in pigs fed NC diet in the present study. However, FA containing sequestering component plus fungal extract and FA containing SMB improved nursery pig growth performance to the level of control. Moreover, tested FA mitigated intestinal oxidative stress through decreased expression of genes for superoxide dismutase, perhaps indicating localized protection of intestinal cells.
